# Adaptive phenotypic variation among clonal ant workers

**DOI:** 10.1098/rsos.170816

**Published:** 2018-02-14

**Authors:** Eisuke Hasegawa, Saori Watanabe, Yuuka Murakami, Fuminori Ito

**Affiliations:** 1 Laboratory of Animal Ecology, Department of Ecology and Systematics, Graduate School of Agriculture, Hokkaido University, Sapporo 060-8589, Japan; 2 Graduate School of Medicine, Department of Neuropharmacology, Hokkaido University, Sapporo 060-8638, Japan; 3 Faculty of Agriculture, Kagawa University, Takamatsu 761-0795, Japan

**Keywords:** clonal reproduction, phenotypic variations, response threshold variance, epigenetics

## Abstract

Phenotypic variations are observed in most organisms, but their significance is not always known. The phenotypic variations observed in social insects are exceptions. Genetically based response threshold variances have been identified among workers and are thought to play several important adaptive roles in social life, e.g. allocating tasks among workers according to demand, promoting the sustainability of the colony and forming the basis of rationality in collective decision-making. Several parthenogenetic ants produce clonal workers and new queens by asexual reproduction. It is not clearly known whether such genetically equivalent workers show phenotypic variations. Here, we demonstrate that clonal workers of the parthenogenetic ant *Strumigenys membranifera* show large threshold variances among clonal workers. A multi-locus genetic marker confirmed that colony members are genetic clones, but they showed variations in their sucrose response thresholds. We examined the changing pattern of the thresholds over time generating hypotheses regarding the mechanism underlying the observed phenotypic variations. The results support the hypothesis that epigenetic modifications that occur after eclosion into the adult form are the cause of the phenotypic variations in this asexual species.

## 1. Introduction

Individual organisms usually show phenotypic variations, so-called ‘individuality’ [1]. Each individual differs from conspecific individuals in several ways, e.g. shape, behaviour, preferences and so on. These individualities are thought to arise from genetic differences in sexual organisms [2]. In group-living animals, phenotypic variations among members play adaptive roles that are essential for survival [3]. Several adaptive roles of phenotypic variation among community members have been elucidated in social insects [4–6].

In social insects, there is variance in the threshold of response to a stimulus. A worker responds to a stimulus when its intensity is higher than the threshold. The response threshold variance among workers has been shown to contribute to several adaptations that are important for social life. First, this phenotypic variation is used among the workforce to allocate tasks according to demand [4]. When a task appears, workers with the lowest thresholds will process it. If additional tasks emerge during task processing, the workers with the next-lowest thresholds will process the new tasks. Thus, a colony can send the required workforce to each task according to demand without the need for a leader [7]. Second, this variance guarantees the sustainability of a colony owing to the inevitable appearance of inactive workers under this system. When diligent workers need to rest due to fatigue, the inactive workers can replace them in crucial tasks that must be processed constantly (e.g. egg licking) [5]. Recently, a third role of this variance has been reported: namely, a basis of rationality in collective decision-making [6]. The threshold variance in the ant *Myrmica kotokui* to concentrations of sucrose solution enables them to choose the better resource [6]. Thus, phenotypic variations among workers are important for survival in colonial life.

Previous studies have shown that the response threshold of a worker is affected by her genotype [8,9]. The existence of many patrilines among honeybee workers has been shown to be adaptive and is mediated by a threshold variance that is caused by underlying genotypic differences [9]. Sexual organisms can create phenotypic variations by mixing genomes from both the mother and the father; however, organisms that asexually reproduce cannot create genetic phenotype variations among their offspring because the children are genetic clones of each other.

In ants, several parthenogenetic species produce diploid daughters without any mating. A dacetine ant, *Strumigenys membranifera* (previously known as *Pyramica membranifera*), is one such parthenogenetic species [10], and all the colony members are thought to be genetic clones [10]. Whether there is an essential threshold variance among such clonal workers and, if it exists, how they create phenotypic variations are important issues that need to be addressed.

There are several studies in which the behavioural variability in clonal ants has been evaluated [11–14]. *Cerapachys biroi* workers have shown the same type of parthenogenetic reproductive mode as *Pristomyrmex punctatus* (previously known as *P. pungens*), in which all workers produce clonal offspring. Thus, in this type of parthenogenetic ant, there are many matrilines that would generate many genetically different workers in a colony by mutations at genome copies in each worker. In fact, a genetic analysis has shown that wild *P. punctatus* colonies are mixtures of multiple genotypes [15]. Although age polyethism has been recognized in wild colonies of a parthenogenetic ant, *Platythyrea punctata* [13], genetic analyses using five microsatellites have shown that most of the wild colonies were mixtures of multiple clones that presumably formed by colony fusions [12]. Irrespective of this genetic evidence, clonality among colony members has not been confirmed in the colonies observed for behavioural patterns of *P. punctata* [13]. Thus, it is ambiguous as to whether clonal individuals have shown the observed behavioural variations in the parthenogenetic species used in the previous studies*.*

In this study, we first examined the clonality among *S. membranifera* workers using a multi-locus genetic marker (a modified fluorescent random amplified polymorphic DNA (RAPD) [16,17]), which is highly suitable for comparing genomic profiles among individuals (for detailed methods, see below and electronic supplementary material, figures S1–S4). Then, we checked the existence of the response threshold variance to sucrose solutions among workers, which has been confirmed as an adaptive trait in rational collective decisions [6]. Finally, we tested three hypotheses of how the variance is generated. The three hypotheses for the determinant of the threshold value of workers are as follows: (1) the threshold value is determined during larval development and does not change after eclosion into the adult form; (2) the threshold value changes in one direction or the other (increases or decreases) over time; and (3) the threshold value changes in random directions owing to experience during the period between the first and second measurements. If hypothesis 1 is the case, then the threshold value would be determined by experience during the larval period (e.g. by nutritional controls as in queen production). If hypothesis 2 is the case, then the threshold value would be determined by some mechanisms (e.g. an accumulation of a chemical substance over time or some developmental process causing a directional change in threshold with age) that would drive it to change in one direction or the other (increase or decrease), and this would produce a threshold variance within a colony because the ages of the workers vary. If hypothesis 3 is the case, then the threshold value would be determined by experiences after eclosion into the adult form, and the most likely cause would be epigenetic modifications (chemical modifications on cytosine (by methylation) or histone) that affect the expression of genes and thus affect the phenotypes. Thus, by examining the pattern of the threshold change, we can estimate the underlying mechanism for threshold variance in this clonal ant.

Based on the results, we will discuss the roles of phenotypic variations in clonal social animals and will propose a hypothesis for the mechanism that generates phenotypic variations in clonal organisms, which should be tested in future studies.

## 2. Material and methods

### 2.1. Study organisms

*Strumigenys membranifera* is a tiny dacetine ant that inhabits urban areas (e.g. parks in towns) and is thus unlike other dacetine ants, most of which inhabit forests [12]. This species has a curious reproductive system. An unmated queen produces diploid female workers. When a colony becomes large, new queens are produced parthenogenetically, and the colony becomes polygynous [10]. Many dissected queens have no sperm in their spermatheca, and virgin queens have been shown to be able to produce diploid daughters without mating [10]. Thus, all the colony members are assumed to be genetic clones.

### 2.2. Colony sampling and rearing

In 2011 and 2016, two colonies were collected from Marugame (Colony KC1) in Kagawa Prefecture and Cape Asizuri (Colony AC1) in Kochi Prefecture in southern Japan, respectively. Official permission was not required to collect these colonies. Each colony was reared in a plastic case (8 × 14 × 3 cm) with a plaster floor. A few collembolans, the first instar larvae of a cricket (*Acheta domesticus*), or adults of a fruit fly species were provided once or twice each week. The colonies were reared at 26°C and with natural light periods.

### 2.3. Examination of clonality between colony members

Clonality among colony members was examined using a modified method with a multi-locus genetic marker (fluorescent RAPD [15]). The used conditions showed high resolution for genomic profiling (see electronic supplementary material, figures S1–S4). Ten workers were randomly selected from KC1, and a pair of workers was selected from AC1 (AC11 and AC12). The total DNA of each individual was extracted using a DNA extraction kit (DNeasy^®^ Blood and Tissue Kit, QIAGEN, Hilden, Germany). Two clonal pairs of aphids (*Macrosiphoniella yomogicola*) were taken from two clonal lines to compare the similarities in the genomic profiles of the two clonal species. As a reference for sexual organisms, pairs of workers were collected from two nests of a monogynous ant, *Pheidole fervida* (collected from the property of Hokkaido University: Pf11 and Pf12 from colony 1 and Pf21 and Pf22 from colony 2). We also used two pairs of workers from two colonies of a monogynous and sexual ant, *Myrmica kotokui* (Mk31–32 and Mk81–82) collected from the Tomakomai experimental forest of Hokkaido University. The total DNA was extracted individually using the same DNA extraction kit. All the DNAs were adjusted to 10 pmol μl^−1^ before amplification. Each DNA sample was amplified by polymerase chain reaction (PCR) using a 10-mer primer (OPA02 or OPA07; Cosmo Genetech, Seoul, South Korea) with a fluorescent marker at the 5′ end. Because the fidelity of amplification of RAPD fragments has been known to increase with annealing temperature in the PCR protocol [16], we selected the optimal annealing temperature from three annealing temperatures (30, 40 or 50°C). We used Ex Taq polymerase (TaKaRa, Shiga, Japan) and the kit buffer for PCR reactions. The composition of the reaction mix was as follows: 0.5 µl of x10 Ex Taq buffer, 0.5 µl of 4dNTP (2.5 mM each), 6 pmol of a labelled primer, 0.15 U of Ex Taq (0.03 µl), 0.5 µl of DNA (5 pmol) and 2.9 µl of sterilized distilled water (the total volume was 5 µl). Thermal cycling was as follows: 2 min at 94°C, 45 cycles consisting of 20 s at 94°C, 20 s at an annealing temperature (30, 40 or 50°C) and 4 min at 60°C. Cycles were conducted in a thermal cycler (2720 Thermal Cycler, Applied Biosystems, MA, USA). The samples were held at 4°C after a thermal cycling until electrophoresis.

The amplified fragments were separated by an automated capillary genetic analyser (CEQ 8000, SCIEX, MA, USA). The presence or absence of a fragment at each length from approximately 50–700 nt was recorded for each sample. The presence or absence of an amplified fragment at each length position was confirmed by a length of 1 nt during the fragment analysis using an automated capillary sequencer (CEQ 8000, SCIEX, MA, USA). Numerous fragments could be detected (see electronic supplementary material, figure S1) because the used size marker (CEQ™ DNA Size Standard Kit-600 (for 60–600 bp)) enabled us to determine the length of amplified fragments from approximately 50–700 nt by 1 nt. We defined the criterion for the presence of a fragment at a length site as follows: when any sample amplified by a primer showed a peak with a fluorescence strength of more than 200 at a given length site, the presence of a fragment of that length was recognized. Then, when another sample had a recognizable peak at that site, we judged the fragment as present at that site, even when the fluorescence strength of the peak was lower than 200. By the above criterion, we determined the genomic profiles of all the samples amplified by each primer. A fragment-sharing rate (which is indicative of similarity between genomic profiles) was calculated for each sample pair for each primer, and then the difference in the fragment-sharing rates between two samples was tested by using chi-square tests.

### 2.4. Examination of the threshold variance within a colony

We examined 37 workers from the KC1 colony and 46 workers from the AC1 colony. All the workers in each nest were marked with 10 colours of a paint (Paint Marker, Mitsubishi) on the thorax and abdomen to enable us to distinguish individuals. We measured the response threshold of each worker to concentrations of sucrose solution. We prepared 0–10% sucrose solutions at 1% intervals. Twenty microlitres of a solution was placed in a plastic Petri dish (5 cm in diameter). A worker was introduced into the dish, and we observed the behaviour of the worker for 1 min after she touched the surface of the solution with her antennae. When she drank the solution within the observation period, we judged that she had responded to the concentration. We investigated the responses of a worker in solutions that varied from a weak solution (0%) to a condensed solution (10%) at 2% intervals. When a worker responded to a concentration, we checked her response to the solution at a 1% lower concentration than the concentration that had elicited a response. For example, when a worker did not respond to 2% but responded to 4%, we checked the response to a 3% solution. In this way, we determined the threshold value for each worker at 1% intervals. All the examined workers responded to the solutions with a more condensed concentration than the lowest concentration to which she responded (e.g. if a worker responded to a 2% solution, she responded to all solutions with a concentration greater than 2%).

### 2.5. Change in the threshold value over time

For the AC1 colony, we re-measured the threshold value of each of the surviving workers after approximately one month to investigate whether the threshold value had changed over time. We proposed three hypotheses regarding how the threshold value of a worker is determined. Hypothesis 1 stated that the threshold value is determined during larval development and does not change after eclosion into the adult form. Hypothesis 2 stated that the threshold value changes in one direction (increases or decreases) over time. Hypothesis 3 stated that the threshold value changes in a random direction owing to experiences that occur during the time between the first and second measurements. If hypothesis 1 is the case, then the threshold value is determined by experiences that occur during the larval period. If hypothesis 2 is the case, then the threshold value is changed by a chemical mechanism (e.g. an accumulation of a chemical substance over time) in one direction (increase or decrease), which can produce threshold variance within a colony because of the varying ages of the workers. If hypothesis 3 is the case, then the threshold value is determined by experiences that occur after eclosion into the adult form, and the most likely cause is epigenetic modifications that affect gene expression and thereby affect the threshold value. Thus, in examining the pattern of the threshold change, we can estimate the underlying mechanism of threshold variance in this clonal ant.

### 2.6. Statistical analyses

All the statistical analyses were conducted using R (v. 3.2.1).

## 3. Results

**Figure 1.**
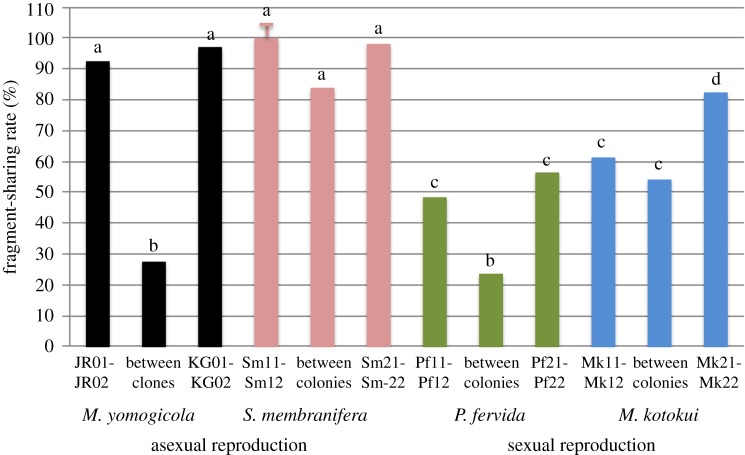
The fragment-sharing rate (which is indicative of similarity between genomic profiles) between pairs of individuals of several sexual and asexual species. When the letters on the bar are the same, there is no significant difference between the rates. When the letters on the bar are different, there is a significant difference between the rates after the Bonferroni correction for multiple comparisons. *Strumigenys membranifera* workers showed a fragment-sharing rate similar to that found between pairs of clonal aphids (*Macrosiphoniella yomogicola*) but significantly higher than that found between nest mates of the sexual and monogynous ants *Pheidole fervida* and *Myrmica kotokui*.

The modified RAPD produced several tens to several hundreds of fragments per primer. At the annealing temperature of 50°C, the fragment-sharing rates (which are indicative of similarity between two genomic profiles) within clones in the clonal aphids were 94.81–100%, and that between clones was 27.78%. This difference was statistically significant (Fisher's exact test, *p* = 0.00236). The fragment-sharing rates among *S. membranifera* nest mates were 99.48–100% in the colony KC1 and 97.89% in the colony AC1, and these values were not significantly different from that within the clonal aphids (96.6–97.4%: Fisher's exact probability test; for all the pairs, the difference is not significant after the Bonferroni correction for multiple comparisons). However, the fragment-sharing rates in clonal pairs were significantly higher than those of the nest mates of a sexual and monogynous ant (figure 1). Thus, the results showed that, at least in these colonies, nest mates of *S. membranifera* are genetic clones of each other, and there is no evidence that different clones intrude into the colonies like in other parthenogenetic ants [13,15]. Another line of evidence supports this view: the colony KC1 included a single queen at the time of collection, and it was reared for more than 4 years in the laboratory of Kagawa University. Only queens lay eggs in this species [10]; all the workers have been replaced by the queen's clonal daughters during this long rearing. Thus, all the colony members should be genetic copies without sequence differences that had occurred during genome copying at reproduction. The results of genetic analyses supported this estimation. Although the fragment-sharing rate was not 100% for some primers, this is likely to reflect genomic differences caused by mutations during genome copying at reproduction because the presence and absence of polymorphisms between clonal individuals have replicability between different thermal cycles and thus are unlikely to be attributable to misamplifications during the PCR process (see electronic supplementary material, figure S3).

In total, we estimated the threshold value of 82 workers from two colonies for different concentrations of sucrose solution. Each of the examined workers had a threshold for a concentration of sucrose solution; i.e. when a worker responded to a concentration, she responded to all concentrations higher than the threshold concentration. However, she did not respond to concentrations lower than the threshold (see electronic supplementary material, tables S1 and S2). There were no exceptions to this pattern. These results demonstrated that each worker of *S. membranifera* has a threshold for responding to different concentrations of sucrose solution.

If there were no mechanism for individual variation in a quantitative trait such as the present response threshold, the threshold would be normally distributed among individuals at any snapshot observation (=the null hypothesis). However, the threshold distributions in both the colonies deviated significantly from normality (Shapiro–Wilk normality test: for KC1, *W* = 0.8316, *p* = 0.00014; for AC1, *W* = 0.7954, *p* = 1.025 × 10^−6^). Thus, there should be at least a cause of the observed variations among clones. The variance in response thresholds for concentrations of sucrose solutions has been confirmed as an adaptive trait for collective decisions in ant colonies [6]. In addition, the frequency distribution of the thresholds was significantly different between the two colonies (Fisher's exact test, *p* = 0.00489).

Because the collembolan population as the food source went extinct soon after arriving at the laboratory of Hokkaido University, and KC1 did not eat the alternative foods (a fruit fly species or the first instar larvae of a house cricket (*Acheta domesticus*)), only the first threshold could be used for this colony. Therefore, we re-measured the thresholds for AC1 only. The threshold variances changed in various directions at the second measurement after approximately one month (figure 3). The number of individuals that had an increased threshold value was not significantly different from the number that had a decreased threshold value (workers with an increased threshold versus workers with a decreased threshold: 3/16 versus 4/16; Fisher's exact test, *p* = 0.6772). Thus, the threshold value changed without a specific tendency in the workers in which the threshold value changed. Thus, hypothesis 2 or 3 is supported because this result suggests that there are some mechanisms by which the threshold will change with the age of workers (see below) or the threshold changed with workers' experiences (presumably by epigenetics).

Seven new workers eclosed from pupae (we could distinguish them from the other colony members because they had no paint mark; see the Material and methods section) during the one-month interval between the two measurements, and none of them responded to the 10% solution at the second measurement, indicating that they had a threshold higher than 10%. This result suggests that there is a possibility that the threshold to sucrose concentrations becomes low with age.

## 4. Discussion

At the time of collection, KC1 contained a single queen, and this colony was reared in the rearing room of Kagawa University for over 4 years. At the time of the experiment, there are three daughter queens of the original queen in the colony, but the long rearing periods should replace all the colony members with offspring of the original queen (i.e. the original workers had died during the rearing at Kagawa University). Thus, KC1 is likely to consist of only clonal individuals (=offspring of the original queen). The results of the modified RAPD analyses showed that workers of *S. membranifera* are genetic clones of each other in both KC1 and AC1 (figure 1). The fragment-sharing rate (which is indicative of similarity between two genomic profiles) among workers was similar to that found between clonal aphids but significantly higher than that found between nest mates of a sexual and monogynous ant (figure 1). Because the used aphids were reared from a stem mother for each clonal line, measuring the similarity of genomic profiles between two individuals is sufficient to obtain the level of the fragment-sharing rate within a clone as a reference. For the same reason, using a small number of samples from the sexual ant as a reference for sexual reproduction is not a problem.

Previously, this variance has been assumed to arise from genetic differences among workers [8,9]. However, clonal individuals have only minimal sequence differences caused by mutations during clonal reproduction. In addition, several studies have noted the necessity of phenotypic variations in clonal organisms for the persistence of their own genetic line over many generations [18,19]. In addition, a previous study has directly shown the adaptive significance of the threshold variance among workers to sucrose concentrations [6].

**Figure 2.**
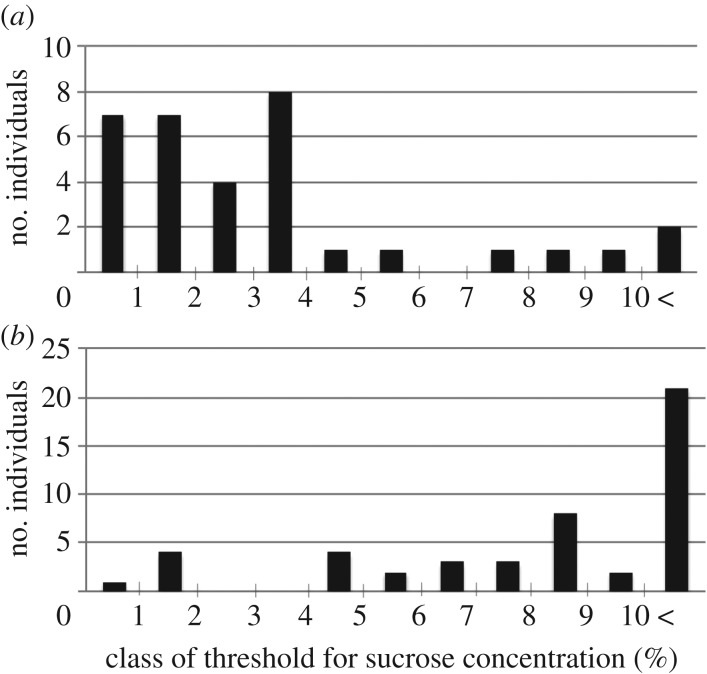
Frequency distributions of the threshold values for concentrations of a sucrose solution in two colonies ((*a*) Marugame colony KC1 and (*b*) Asizuri colony AC1) of the clonal ant *Strumigenys membranifera*. There are considerable variations in the threshold values of workers even though they are genetic clones. When comparing the two colonies, there is a significant difference in the frequency distributions between the two colonies (Fisher's exact test, *p* = 0.00489).

We made hypotheses regarding the three mechanisms that may enable phenotypic variations among clonal workers. Hypothesis 1 stated that the threshold value is determined during the course of larval development by nutritional conditions, as in queen production in the honeybee [20]. If this is correct, then threshold values do not change over time. Hypothesis 2 stated that the threshold value changes in one direction or another (increases or decreases) with age. If an accumulation (or consumption) of a chemical substance controls the threshold value based on age, then this pattern is expected. Hypothesis 3 stated that epigenetic modifications resulting from experiences that occur after eclosion into the adult form change the threshold value in random directions. If this is correct, then the threshold values will change in random directions depending on each worker's experiences. Figure 2 shows that the prediction of hypothesis 3 seems to be the case.

Our results also demonstrate that an adaptive phenotypic variation exists even within clonal ant colony members. Variation in a response threshold can underlie task allocation [4], a colony's long-term sustainability [5] and rational collective decision-making [6]. Note that while the threshold variance can allow rational collective decisions [6], its necessity in collective decision-making in group-living animals is not clear at present. Further studies are needed to clarify this point.

A similar phenomenon, ‘phenotypic plasticity’, has been known to occur [20], in which a genotype develops into different phenotypes under different environments. Several mechanisms of the causes of this phenomenon have been proposed [21]. The observed changes in the thresholds might be a type of phenotypic plasticity. Further studies are required to elucidate the relationship between ‘phenotypic plasticity’ and the current results. The observed phenotypic variation in clonal *S. membranifera* workers may be similarly adaptive for this species, but it is likely to be generated by a mechanism that is independent of genomic identity (see below), although we did not show its importance in *S. membranifera* in this study. However, there are several other mechanisms that can change the thresholds, e.g. learning or neural plasticity. Regardless, future work should elucidate the cause of the observed changes in thresholds.

There are a few studies that investigate divisions of labour in parthenogenetic ants [12,14]. In *Cerapachys biroi*, individual experiences resulted in a lasting division of labour [12], but this species has lost its queen, and all workers can reproduce their own clonal daughters as can another parthenogenetic ant, *Pristomyrmex punctatus*. Although [12] did not check for genetic clonality among the examined workers, the existence of mixed colonies consisting of multiple genetic lines has been confirmed in *P. pungens* [15]. Thus, whether the observed phenotypic variations in *C. biroi* can be completely attributed to differences in experience is unclear. In the other case, a parthenogenetic ant, *Platyrea punctata*, displays age polyethism [14], but this study did not check for genetic clonality among workers, although wild colonies of this species are frequently a mixed group of multiple clones [13]. In addition, the genetic clonality of *P. punctata* was checked by using only five single-locus markers [13], which is less efficient in comparing genomic profiles between individuals than a multi-locus marker with high fidelity and polymorphisms. Therefore, our study is one of the best that showed phenotypic variations among clonal ant workers to date.

The frequency distribution of the thresholds was significantly different between the two colonies (Fisher's exact test, *p* = 0.00489). This difference is interesting. A possibility is that this difference reflected the difference in age compositions between the colonies if the threshold changed with age (see below). Another possibility is that the optimal threshold distribution is different between the populations. Recently, it has been shown that the performance of collective decision by using only yes/no decision units is maximized when the threshold distribution of a collective decision-maker is matched with the distribution of the options' qualities [22]. This issue is worth examining in future studies.

**Figure 3.**
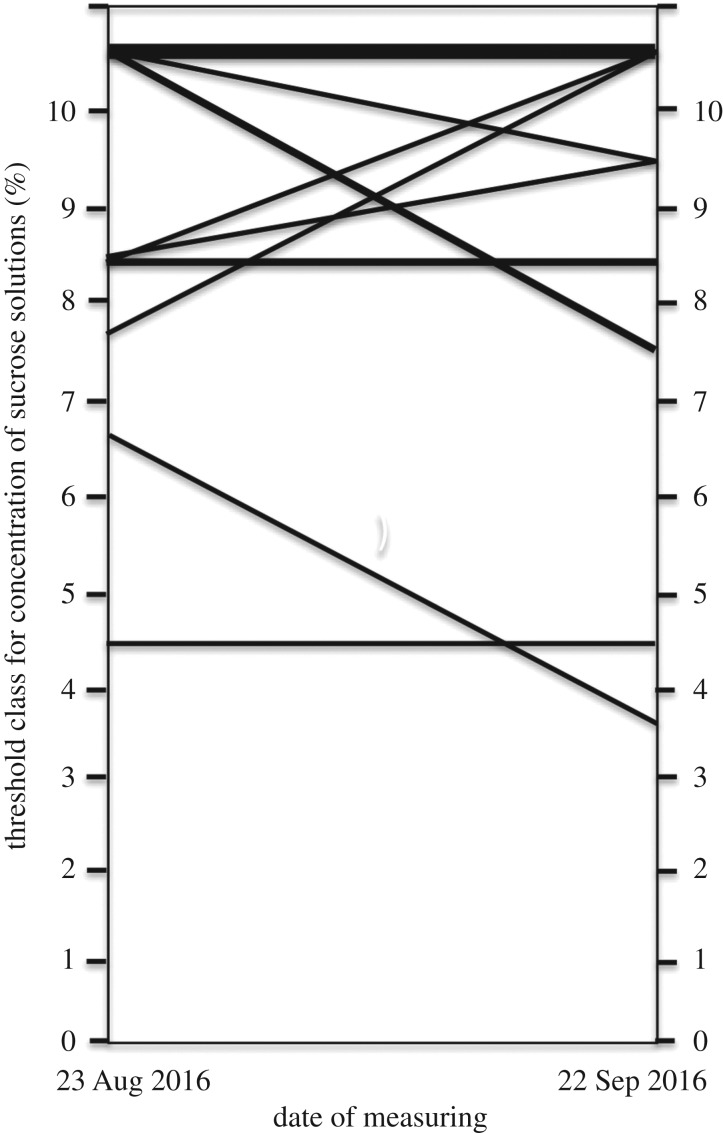
Change in the threshold values between the two measurements taken at approximately a one-month interval. The threshold values of several workers changed, but the direction of the change was not biased from 1 : 1 (increase versus decrease = 3/16 versus 4/16; Fisher's exact test, *p* = 1.000).

Our results showed that the thresholds of several workers changed (figure 3), but those of 9 of 16 workers did not change (figure 3). Previously, thresholds of workers were assumed to be determined genetically [8]. Thus, interesting questions are how the threshold changes and how many are flexible. A recent focus on how one genome can lead to many phenotypes has led to investigations of epigenetic modifications of gene expression [23,24]. Epigenetics has been defined as modulations of gene expressions by chemical modifications on genes (methylation for cytosine in nucleotides or for histone [24]). Epigenetic modifications might explain the observed variance in the threshold value of *S. membranifera*. Previous studies have shown that the threshold value is affected by genotype [8,9], meaning that the genes that control the threshold value are somewhere in a genome. Thus, when these genes are modified epigenetically, a clonal social organism can create threshold variations among members, and this is essential for maintaining the social life of clonal social animals [3–6]. When we identify the genes, we will be able to investigate the epigenetic modifications in *S. membranifera* workers depending on the experiences of individuals [12]. This subject awaits further examination.

Finally, there is the possibility that the observed phenotypic variations come from a combination of the different mechanisms mentioned above. For example, the threshold value may constantly increase or decrease with age (as suggested by the existence of age polyethism in a parthenogenetic ant [14]), and epigenetic modifications caused by individual experiences may randomly affect the direction of change. In *P. membranifera*, all newly eclosed workers (*n* = 7) belonged to the highest threshold class (greater than 10%). This fact suggests that the threshold to sucrose concentration is highest at eclosion and gradually decreases with age, but individual experiences affect the threshold value epigenetically in random directions during the course of the gradual change, resulting in a highly varied threshold distribution within a colony (figure 2*a*,*b*). This study demonstrates the existence of adaptive phenotypic variations within a clonal colony of a parthenogenetic ant. Further studies are required to elucidate how the colony creates phenotypic variations in its members, and these studies will bring many new insights into the reasons for the existence of phenotypic variations (that is, individual diversity) in organisms [1].

## Supplementary Material

Variances in a clonal ant OpenSci 1st Rev Supplementary

## Data accessibility

All the data used in this study have been uploaded to Dryad Digital Repository (http://dx.doi.org/10.5061/dryad.nj1qv) [25].
