# Supplementary material for: Adaptive phenotypic variation among clonal ant workers
Source: R Soc Open Sci. 2018 Feb 14;5(2):170816. doi: 10.1098/rsos.170816 (PMC5830712; doi:10.1098/rsos.170816)
Supplement: Variances in a clonal ant OpenSci 1st Rev Supplementary [file rsos170816supp1.docx]

**Supplementary materials**

Title: An adaptive phenotypic variation among clonal ant workers.

Authors: Eisuke Hasegawa, Saori Watanabe, Yuuka Murakami and Fuminori Ito.

**Supplementary methods: *Experimental procedure of a modified RAPD***

We synthesized 6 RAPD primers (OPA02, 03, 08, 10, and 20, the same sequence as the Oeron primers provided by Eurofins Genomics, Tront, Canada) that were labeled with one of the Beckman-dyes at the 5'-end. We extracted total DNA from 2 individuals from 2 monoclonal lines of the aphid, *Macrocyphoniella yomogicola*, by using a DNA extraction kit (QIAGEN, DNeasy^Ⓡ^ Blood and Tissue Kit, QIAGEN, Hilden, Germany). DNA was eluted to 200 µl in the buffer (buffer AE) from the kit. We used Ex-taq polymerase (TaKaRa, Shiga, Japan) and the kit buffer for PCR reactions. The composition of the reaction mix was as follows: 0.5 µl of x10 Ex taq buffer, 0.5 μl of 4dNTP (2.5 mM each), 6 pmol of a labeled primer, 0.15 U of Ex Taq (0.03 μl), 0.5 μl of DNA, and 2.9 μl of sterilized distilled water (the total volume was 5 μl). Thermal cycling was as follows: 2 min at 94℃, 45 cycles consisting of 20 sec at 94℃, 20 sec at an annealing temperature (30, 40, or 50℃) and 4 min at 60℃. Cycles were conducted in a thermal cycler (2720 Thermal Cycler, Applied Biosystems, MA, USA). The samples were held at 4℃ after a thermal cycling until electrophoresis. The amplified fragments were separated by using an automated capillary genetic analysis system (CEQ8000, Sciex, Ontario, Canada). A volume of 1.5 μl from each reaction was loaded in 39.5 µl of Sample loading solution (GenomLab Sample Loading Solution (SLS), Beckman Coulter, CA, USA) with 0.5 μl of a size marker covering 50~600 bp (GenomLab DNA Size Standard Kit-600, Beckman Coulter, CA, USA). Data were analyzed by using the fragment analysis software of the system.

The presence or absence of a fragment at each length position was determined by the 1 nt length. Numerous fragments could be detected (Figure S1A-C) because the size marker enabled the determination of the length of fragments from ca. 50 to 700 nt by 1 nt. We defined the criterion for the presence of a fragment at a length-site as follows: when a sample showed a peak with a fluorescent strength of more than 200 at a given length, we defined the presence of a fragment of that length. Then, when another sample had a recognizable peak at that site, we judged the presence of the fragment at that length, even when the fluorescent strength of the peak was lower than 200.

*Determination of annealing temperature for reliable amplification*

The original RAPD uses a very low annealing temperature (35℃). The Tm temperatures are low for such a short primer, but this low annealing temperature might be a cause of the low fidelity of RAPD amplifications [*17*]. Therefore, we tested three annealing temperatures, 30℃, 40℃, and 50℃. For each annealing temperature, we examined the fragment-sharing rate of the products of OPA02 between two clonal individuals of *M. yomogicola* (JR01 and JR02)*.*

*Repeatability of amplification*

For several primers, we examined the repeatability of fragment amplifications by using the same DNA and the same thermal cycle settings in the same thermal cycler. In the 1st cycle, fragments from two clonal pairs (JR01 and JR 02, KG01 and KG02) of *M. yomgicola* were amplified with OPA 02, and in the 2nd cycle, each sample was amplified in the same way as the 1st cycle in 4 different tubes. Then, the fragment-sharing rates were calculated for each pair of tubes.

*Inheritance manner of fragments,*

We reared a male and a female *Acheta domestica* cricket from larvae under solitary conditions. When they eclosed to adults, they were reared together in a plastic container (15 x 20 x 10 cm), and copulation was confirmed. The female was allowed to oviposit on wet cotton at 26℃, and several days later (to allow cell divisions of eggs to extract a sufficient amount of DNA to amplify), the cotton was preserved in acetone for subsequent egg DNA preparation. DNA was extracted from the eggs and the thorax muscles of the parents, using a DNA extraction kit (QIAGEN, DNeasy^Ⓡ^ Blood and Tissue Kit, QIAGEN, Hilden, Germany). DNA was eluted in the kit buffer in volumes of 200 µl for the parents and 50 µl for the eggs. We obtained DNA from 18 eggs. Then, each DNA sample was amplified with the same cycle parameters, with a 50℃ annealing temperature and the thermal cycle described above. Then, amplified fragments from each DNA sample were separated and examined for the presence or absence of fragments at each length position. Then, the fragment segregation ratios among the eggs were calculated for each position where a fragment was detected for either of the parents. Because RAPD fragments are dominant (cannot discriminate homozygote and heterozygote when a fragment present at a site), three segregation ratios were expected under Mendelian inheritance. When the mother or father is homozygote at a site, all of the offspring have the fragment; in other words, the segregation ratio should be 1.0. When both parents are heterozygotes, the segregation ratio should be 3:1 for presence:absence in the offspring. When the mother or father is heterozygous and the other parent does not have the fragment at a site, the segregation rate should be 1:1 for the presence vs. absence within the offspring. Therefore, when complete Mendelian inheritance occurs, the segregation rates at each site should be 1.0, 0.75 or 0.5.

However, because the numbers of examined offspring were small (less than 18), the observed segregation rate can deviate from the value expected by chance. For example, if 10 eggs were examined, theoretically, the segregation ratio should be 0.5 (=5:5) in the case of a heterozygote x null site; in practice, however, it could be 6:4, 7:3 or even 10:0 by chance. Therefore, we conducted a simulation to predict the expected frequency distribution of segregation ratios for each primer and compared the observed distributions with the expected one. When the fragments were inherited in a Mendelian manner, the distributions (observed and predicted) should not differ significantly.

To estimate the expected distribution, we conducted simulations in which we assigned presence or absence of a fragment at a length-site on the basis of a probability (p=0.5 or 0.75). A random number (n) was generated, and if n<p, the presence of a fragment was assumed at that site. The estimated distribution was influenced by the numbers of sites with p=0.5 or 0.75. Thus, we needed to estimate the number of sites with either of these segregation rates. Because the 0.5 segregation rate occurs when either the mother or father has a fragment and the other parent is null, we counted the number of such sites and subtracted the number of sites with a segregation rate of 1.0, which indicates that either of the parents is homozygous. Similarly, a segregation rate of 0.75 occurs only when both parents are heterozygous. Therefore, to estimate the number of types of sites, we counted the number of sites at which both parents had a fragment with a segregation rate among the offspring that was not 1.0. For each site from each examined primer (OPA02, 03 and 07), we assigned presence or absence on the basis of the above site type to the number of eggs for which data could be obtained for each primer (9 for OPA02, 13 for OPA03 and 14 for OPA07). The simulation was repeated 100 times for each primer, and on the basis of these simulations, we made an estimated frequency distribution by summing the average number of segregation rates for all sites for each primer. The number of the sites with a rate of 1.0 was compared with the number of observed sites with ratios of 1.0. Then, we compared the frequency distribution of segregation rates (0~1.0 in increments of 0.1) between the observed and expected distributions by a χ2-test. In some cases, no fragment was amplified for some egg DNA samples, or the results of electrophoreses did not allow us to read the full range (ca. 50-700 nt) of fragment length. Thus, we used a total of 122, 395 and 133 fragment sites for OPA02, 03 and 07, respectively.

*Degree of polymorphism*

To examine the degree of polymorphism, we collected 2 individuals each from two different nests of the monogynous ants, *Pheidole ferivida* and *Myrmica kotokui*, from the property of Hokkaido University and the Tomakomai experimental forest of Hokkaido university, respectively. In total, 2 individuals from two different colonies (in total, 4 individuals for a species) were collected. Total DNA was extracted with a DNA extraction kit (QIAGEN, DNeasy^Ⓡ^ Blood and Tissue Kit, QIAGEN, Hilden, Germany) and eluted in 200 μl of the kit buffer. Each DNA was amplified with OPA02 and OPA07, and the amplified fragments were analyzed by the procedure described above. Then, we calculated the fragment-sharing rates within a colony and between colonies for both species to estimate the degree of polymorphism of RAPD fragments.

**Results and Figure legends**

Figure S1. The results of electrophoresis for the products of 3 primers. Many fragments were amplified by fluorescence-labeled 10-mer primers. The red peaks are from the size marker.

Figure S2. The relationship between annealing temperature and fragment-sharing rate between two clonal individuals of the aphid *Macrocyphonielle yomogicola* (JR01-JR02). A low temperature resulted in low sharing rates. However, 50℃ annealing showed 96.4% sharing. The remaining 3.6% of the polymorphic sites appeared to represent genetic diversification between two individuals, which occurred during multiple clonal reproductions (see Fig. 3).

Figure S3. Consistent polymorphisms between two clonal aphids. The upper panels (A &B) represent the results of amplification using OPA02 for JR01 (A) and JR02(B). Three fragments (478, 479 and 480 nt) were present in JR01 but are absent in JR02 (indicated by red arrows). In the 2nd amplification, the same results were observed (C & D). Therefore, these polymorphisms appeared to represent genetic differences between the two individuals.

Figure S4. Segregation analyses for each fragment from 3 primers (OPA02: upper, 03: middle, 07: lower) from a pair of a virgin female and a male cricket (*Acheta domestica*) and their offspring. The left 3 panels are the observed frequency distributions of segregation rates, and the right panels are the expected ones. We used data from 9, 13 and 14 offspring eggs for primers OPA02, 03 and 07, respectively. For all 3 primers, the distribution did not differ significantly (for statistical tests, see text), thus suggesting that each fragment was inherited in a Mendelian manner.

Figure 1

Figure 2

Figure 3

Figure 4

Table S1. Responses to different concentrations of sucrose solution (0-10%) of workers in the colony KC1 of *Strumigenys membranifera.* Thr. means the estimated threshold value. Zero and 1 mean “did not drink” and “drink”, respectively.

| No. | 0% | 1% | 2% | 3% | 4% | 5% | 6% | 7% | 8% | 9% | 10% | Thr. |
| --- | --- | --- | --- | --- | --- | --- | --- | --- | --- | --- | --- | --- |
| 1 | 0 | 0 | 0 | 1 | 1 | - | 1 | - | 1 | - | 1 | 2.5 |
| 2 | 0 | 0 | 0 | 0 | 0 | 0 | 0 | 0 | 1 | 1 | 1 | 7.5 |
| 3 | 0 | 0 | 0 | 0 | 0 | 0 | 1 | 1 | 1 | - | 1 | 5.5 |
| 4 | 0 | 0 | 0 | 0 | 1 | 1 | 1 | - | 1 | - | 1 | 3.5 |
| 5 | 0 | 0 | 0 | 1 | 1 | - | 1 | - | 1 | - | 1 | 2.5 |
| 6 | 0 | 0 | 0 | 0 | 1 | 1 | 1 | - | 1 | - | 1 | 3.5 |
| 7 | 0 | 0 | 1 | 1 | 1 | - | 1 | - | 1 | - | 1 | 1.5 |
| 8 | 0 | 0 | 0 | 0 | 0 | 0 | 0 | 0 | 0 | 0 | 1 | 9.5 |
| 9 | 0 | 1 | 1 | - | 1 | - | 1 | - | 1 | - | 1 | 0.5 |
| 10 | 0 | 0 | 0 | 0 | 1 | 1 | 1 | - | 1 | - | 1 | 3.5 |
| 11 | 0 | 0 | 1 | 1 | 1 | - | 1 | - | 1 | - | 1 | 1.5 |
| 12 | 0 | 0 | 0 | 0 | 1 | 1 | 1 | - | 1 | - | 1 | 3.5 |
| 13 | 0 | 0 | 0 | 0 | 0 | 0 | 0 | 0 | 0 | 0 | 0 | 10.5 |
| 14 | 1 | - | 1 | - | 1 | - | 1 | - | 1 | - | 1 | 0 |
| 15 | 0 | 0 | 1 | 1 | 1 | - | 1 | - | 1 | - | 1 | 1.5 |
| 16 | 0 | 0 | 0 | 0 | 0 | 1 | 1 | - | 1 | - | 1 | 4.5 |
| 17 | 0 | 0 | 1 | 1 | 1 | - | 1 | - | 1 | - | 1 | 1.5 |
| 18 | 0 | 0 | 0 | 0 | 0 | 0 | 0 | 0 | 0 | 0 | 0 | 10.5 |
| 19 | 0 | 0 | 0 | 0 | 1 | 1 | 1 | - | 1 | - | 1 | 3.5 |
| 20 | 0 | 1 | 1 | - | 1 | - | 1 | - | 1 | - | 1 | 0.5 |
| 21 | 0 | 0 | 1 | 1 | 1 | - | 1 | - | 1 | - | 1 | 1.5 |
| 22 | 0 | 0 | 1 | 1 | 1 | - | 1 | - | 1 | - | 1 | 1.5 |
| 23 | 0 | 0 | 0 | 0 | 1 | 1 | 1 | - | 1 | - | 1 | 3.5 |
| 24 | 0 | 0 | 0 | 0 | 1 | 1 | 1 | - | 1 | - | 1 | 3.5 |
| 25 | 0 | 1 | 1 | - | 1 | - | 1 | - | 1 | - | 1 | 0.5 |
| 26 | 0 | 0 | 0 | 1 | 1 | - | 1 | - | 1 | - | 1 | 2.5 |
| 27 | 0 | 0 | 1 | 1 | 1 | - | 1 | - | 1 | - | 1 | 1.5 |
| 28 | 0 | 0 | 0 | 1 | 1 | - | 1 | - | 1 | - | 1 | 2.5 |
| 29 | 0 | 1 | 1 | - | 1 | - | 1 | - | 1 | - | 1 | 0.5 |
| 30 | 0 | 0 | 0 | 0 | 1 | 1 | 1 | - | 1 | - | 1 | 3.5 |
| 31 | 0 | 1 | 1 | - | 1 | - | 1 | - | 1 | - | 1 | 0.5 |
| 32 | 1 | 1 | 1 | - | 1 | - | 1 | - | 1 | - | 1 | 0 |
| 33 | 0 | 0 | 0 | 0 | 0 | 0 | 0 | 0 | 0 | 1 | 1 | 8.5 |

Table S2. Responses to different concentrations of sucrose solution (0-10%) of workers in the colony AC1 of *Strumigenys membranifera.* Thr. means the estimated threshold value. Zero and 1 mean “did not drink” and “drink”, respectively.

| No. | 0% | 1% | 2% | 3% | 4% | 5% | 6% | 7% | 8% | 9% | 10% | Thr. |
| --- | --- | --- | --- | --- | --- | --- | --- | --- | --- | --- | --- | --- |
| 1 | 0 | 0 | 1 | 1 | 1 | - | 1 | - | 1 | - | 1 | 1.5 |
| 2 | 0 | 1 | 1 | - | 1 | - | 1 | - | 1 | - | 1 | 0.5 |
| 3 | 0 | 0 | 1 | 1 | 1 | - | 1 | - | 1 | - | 1 | 1.5 |
| 4 | 0 | 0 | 1 | 1 | 1 | - | 1 | - | 1 | - | 1 | 1.5 |
| 5 | 0 | 0 | 1 | 1 | 1 | - | 1 | - | 1 | - | 1 | 1.5 |
| 6 | 0 | 0 | 0 | 0 | 0 | 1 | 1 | - | 1 | - | 1 | 4.5 |
| 7 | 0 | 0 | 0 | 0 | 0 | 1 | 1 | - | 1 | - | 1 | 4.5 |
| 8 | 0 | 0 | 0 | 0 | 0 | 0 | 1 | 1 | 1 | - | 1 | 5.5 |
| 9 | 0 | 0 | 0 | 0 | 0 | 0 | 0 | 1 | 1 | - | 1 | 6.5 |
| 10 | 0 | 0 | 0 | 0 | 0 | 0 | 0 | 1 | 1 | - | 1 | 6.5 |
| 11 | 0 | 0 | 0 | 0 | 0 | 0 | 0 | 0 | 1 | 1 | 1 | 7.5 |
| 12 | 0 | 0 | 0 | 0 | 0 | 0 | 0 | 0 | 1 | 1 | 1 | 7.5 |
| 13 | 0 | 0 | 0 | 0 | 0 | 0 | 0 | 1 | 1 | - | 1 | 6.5 |
| 14 | 0 | 0 | 0 | 0 | 0 | 0 | 0 | 1 | 1 | - | 1 | 6.5 |
| 15 | 0 | 0 | 0 | 0 | 0 | 0 | 0 | 0 | 1 | 1 | 1 | 7.5 |
| 16 | 0 | 0 | 0 | 0 | 0 | 0 | 0 | 0 | 1 | 1 | 1 | 7.5 |
| 17 | 0 | 0 | 0 | 0 | 0 | 0 | 0 | 0 | 1 | 1 | 1 | 7.5 |
| 18 | 0 | 0 | 0 | 0 | 0 | 0 | 0 | 0 | 0 | 1 | 1 | 8.5 |
| 19 | 0 | 0 | 0 | 0 | 0 | 0 | 0 | 0 | 0 | 1 | 1 | 8.5 |
| 20 | 0 | 0 | 0 | 0 | 0 | 0 | 0 | 0 | 0 | 1 | 1 | 8.5 |
| 21 | 0 | 0 | 0 | 0 | 0 | 0 | 0 | 0 | 0 | 1 | 1 | 8.5 |
| 22 | 0 | 0 | 0 | 0 | 0 | 0 | 0 | 0 | 0 | 0 | 1 | 9.5 |
| 23 | 0 | 0 | 0 | 0 | 0 | 0 | 0 | 0 | 0 | 0 | 1 | 9.5 |
| 24 | 0 | 0 | 0 | 0 | 0 | 0 | 0 | 0 | 0 | 1 | 1 | 8.5 |
| 25 | 0 | 0 | 0 | 0 | 0 | 0 | 0 | 0 | 0 | 1 | 1 | 8.5 |
| 26 | 0 | 0 | 0 | 0 | 0 | 0 | 0 | 0 | 0 | 1 | 1 | 8.5 |
| 27 | 0 | 0 | 0 | 0 | 0 | 0 | 0 | 0 | 0 | 1 | 1 | 8.5 |
| 28 | 0 | 0 | 0 | 0 | 0 | 0 | 0 | 0 | 0 | 0 | 0 | 10.5 |
| 29 | 0 | 0 | 0 | 0 | 0 | 0 | 0 | 0 | 0 | 0 | 0 | 10.5 |
| 30 | 0 | 0 | 0 | 0 | 0 | 0 | 0 | 0 | 0 | 0 | 0 | 10.5 |
| 31 | 0 | 0 | 0 | 0 | 0 | 0 | 0 | 0 | 0 | 0 | 0 | 10.5 |
| 32 | 0 | 0 | 0 | 0 | 0 | 0 | 0 | 0 | 0 | 0 | 0 | 10.5 |
| 33 | 0 | 0 | 0 | 0 | 0 | 0 | 0 | 0 | 0 | 0 | 0 | 10.5 |
| 34 | 0 | 0 | 0 | 0 | 0 | 0 | 0 | 0 | 0 | 0 | 0 | 10.5 |
| 35 | 0 | 0 | 0 | 0 | 0 | 0 | 0 | 0 | 0 | 0 | 0 | 10.5 |
| 36 | 0 | 0 | 0 | 0 | 0 | 0 | 0 | 0 | 0 | 0 | 0 | 10.5 |
| 37 | 0 | 0 | 0 | 0 | 0 | 0 | 0 | 0 | 0 | 0 | 0 | 10.5 |
| 38 | 0 | 0 | 0 | 0 | 0 | 0 | 0 | 0 | 0 | 0 | 0 | 10.5 |
| 39 | 0 | 0 | 0 | 0 | 0 | 0 | 0 | 0 | 0 | 0 | 0 | 10.5 |
| 40 | 0 | 0 | 0 | 0 | 0 | 0 | 0 | 0 | 0 | 0 | 0 | 10.5 |
| 41 | 0 | 0 | 0 | 0 | 0 | 0 | 0 | 0 | 0 | 0 | 0 | 10.5 |
| 42 | 0 | 0 | 0 | 0 | 0 | 0 | 0 | 0 | 0 | 0 | 0 | 10.5 |
| 43 | 0 | 0 | 0 | 0 | 0 | 0 | 0 | 0 | 0 | 0 | 0 | 10.5 |
| 44 | 0 | 0 | 0 | 0 | 0 | 0 | 0 | 0 | 0 | 0 | 0 | 10.5 |
| 45 | 0 | 0 | 0 | 0 | 0 | 0 | 0 | 0 | 0 | 0 | 0 | 10.5 |
| 46 | 0 | 0 | 0 | 0 | 0 | 0 | 0 | 0 | 0 | 0 | 0 | 10.5 |
| 47 | 0 | 0 | 0 | 0 | 0 | 0 | 0 | 0 | 0 | 0 | 0 | 10.5 |
| 48 | 0 | 0 | 0 | 0 | 0 | 0 | 0 | 0 | 0 | 0 | 0 | 10.5 |
| 49 | 0 | 0 | 0 | 0 | 0 | 0 | 0 | 0 | 0 | 0 | 1 | 9.5 |
